# Combined Prophylactic and Therapeutic Use Maximizes Hydroxychloroquine Anti-SARS-CoV-2 Effects *in vitro*

**DOI:** 10.3389/fmicb.2020.01704

**Published:** 2020-07-10

**Authors:** Nicola Clementi, Elena Criscuolo, Roberta Antonia Diotti, Roberto Ferrarese, Matteo Castelli, Lorenzo Dagna, Roberto Burioni, Massimo Clementi, Nicasio Mancini

**Affiliations:** ^1^Laboratory of Microbiology and Virology, Vita-Salute San Raffaele University, Milan, Italy; ^2^Laboratory of Microbiology and Virology, IRCCS San Raffaele Scientific Institute, Milan, Italy; ^3^Unit of Immunology, Rheumatology, Allergy and Rare Diseases (UnIRAR), IRCCS San Raffaele Scientific Institute, Vita-Salute San Raffaele University, Milan, Italy

**Keywords:** hydroxychloroquine, SARS-CoV-2, COVID-19, prophylaxis, therapy

## Abstract

While the SARS-CoV-2 pandemic is heavily hitting the world, it is of extreme importance that significant *in vitro* observations guide the quick set up of clinical trials. In this study, we evidence that the anti-SARS-CoV2 activity of a clinically achievable hydroxychloroquine concentration is maximized only when administered before and after the infection of Vero E6 and Caco-2 cells. This suggests that only a combined prophylactic and therapeutic use of hydroxychloroquine may be effective in limiting viral replication in patients.

## Introduction

On 11th March 2020, WHO Director-General characterized COVID-19 as a pandemic. As of 16th June 2020, 7,941,791 total cases were confirmed, accounting for 434,796 deaths (Dong et al., 2020). To date, the clinical management of COVID-19 subjects almost exclusively consists of supportive therapy and, in particular, of ventilatory support for the most severe cases. Several drugs, some already in clinical trials, are currently being used in order to limit the inflammatory response or to hamper SARS-CoV-2 replication (Tu et al., 2020). Regarding the latter, no SARS-CoV-2-specific drugs are currently available and, as a consequence, there is the need for repurposing drugs used in other settings, such as chloroquine (CQ) and hydroxychloroquine (HCQ) (Gao et al., 2020; Gautret et al., 2020; Zhou et al., 2020). Several studies already demonstrated the *in vitro* efficacy of CQ and HCQ, evidencing the latter's higher activity on SARS-CoV-2 (Colson et al., 2020; Wang et al., 2020). Importantly, a recent study also modeled the main pharmacokinetic features of HCQ trying to infer its lung concentration after different dosing regimens (Yao et al., 2020).

It is therefore important to use this data in order to set up possible scenarios related to the real clinical use of this drug. In this study, we tested HCQ against a SARS-CoV-2 Italian clinical isolate, by using different protocols of drug administration corresponding to its possible prophylactic, therapeutic, and prophylactic/therapeutic use in patients. A single HCQ concentration easily reachable in the lung and characterized by high anti-SARS-CoV-2 activity was used for the three protocols (Liu et al., 2020; Wang et al., 2020; Yao et al., 2020).

## Methods

### Cells, Virus, and Antivirals

Vero E6 (Vero C1008, clone E6—CRL-1586; ATCC) cells were cultured in Dulbecco's Modified Eagle Medium (DMEM) supplemented with non-essential amino acids (NEAA, 1x), penicillin/streptomycin (P/S, 100 U/mL), HEPES buffer (10 mM) and 10% (v/v) Fetal bovine serum (FBS). Caco-2 (Human epithelial colorectal adenocarcinoma cells, ATCC HTB-37) cells were cultured in Minimum Essential Medium (MEM) supplemented with NEAA (1x), P/S (100 U/mL), HEPES buffer (10 mM), sodium pyruvate (1 mM), and 20% (v/v) FBS. Calu-3 (Human lung cancer cell line, ATCC HTB-55) were cultured in MEM supplemented with NEAA (1x), P/S (100 U/mL), HEPES buffer (10 mM), and 10% (v/v) FBS. A clinical isolate hCoV-19/Italy/UniSR1/2020 (GISAID accession ID: EPI_ISL_413489) was isolated and propagated in Vero E6 cells, and viral titer was determined by 50% tissue culture infective dose (TCID_50_) and plaque assay for confirming the obtained titer. All the infection experiments were performed in a biosafety level-3 (BLS-3) laboratory of Microbiology and Virology at Vita-Salute San Raffaele University, Milan, Italy. Bafilomycin A1 (BFLA1) and Hydroxychloroquine (HCQ) were obtained from Merck.

### Virus Isolation

An aliquot (0.8 mL) of the transport medium of the nasopharyngeal swab (COPAN's kit UTM® universal viral transport medium—COPAN) of a mildly symptomatic SARS-CoV-2 infected patient was mixed with an equal volume of DMEM without FBS and supplemented with double concentration of P/S and Amphotericin B. The mixture was added to 80% confluent Vero E6 cells monolayer seeded into a 25 cm^2^ tissue culture flask. After 1 h adsorption at 37°C, 3 mL of DMEM supplemented with 2% FBS and Amphotericin B were added. Twenty-four hours post-infection (hpi) another 2 mL of DMEM supplemented with 2% FBS and Amphotericin B were added. Live images were acquired (Olympus CKX41 inverted phase-contrast microscopy) daily for evidence of cytopathic effects (CPE), and aliquots were collected for viral RNA extraction and In-house one-step real-time RT-PCR assay (10.1016/ S0140-6736(20)30154-9). Five days post-infection (dpi) cells and supernatant were collected, aliquoted, and stored at −80°C (P1). For secondary (P2) virus stock, Vero E6 cells seeded into 25 cm^2^ tissue culture flasks were infected with 0.5 mL of P1 stored aliquot, and infected cells and supernatant were collected 48 hpi and stored at −80°C. For tertiary (P3) virus stock, Vero E6 cells seeded into 75 cm^2^ tissue culture flasks were infected with 1.5 mL of P2 stored aliquot and prepared as above described.

### Virus Titration

P3 virus stocks were titrated using both Plaque Reduction Assay (PRA, PFU/mL) and Endpoint Dilutions Assay (EDA, TCID_50_/mL). For PRA, confluent monolayers of Vero E6 cells were infected with 10-fold-dilutions of virus stock. After 1 h of adsorption at 37°C, the cell-free virus was removed. Cells were then incubated for 46 h in DMEM containing 2% FBS and 0.5% agarose. Cells were fixed and stained, and viral plaques were counted. For EDA, Vero E6 cells (4 × 10^5^ cells/mL) were seeded into 96 wells plates and infected with base 10 dilutions of virus stock. After 1 h of adsorption at 37°C, the cell-free virus was removed, and complete medium was added to cells. After 48 h, cells were observed to evaluate CPE. TCID_50_/mL was calculated according to the Reed–Muench method.

### Sequence Analysis

Viral genome from supernatant infected cells was extracted using QIAamp Viral RNA Mini Kit following manufacturers' instructions. Reverse transcription and subsequent amplification were performed using random hexamer primers. The amplicons were sequenced on the Illumina MiSeq NGS platform (Illumina, San Diego, CA, USA). Amplicon purification and quantification were performed by Agencourt AMPure XP (Beckman Coulter, Villepinte, France) and Qubit dsDNA Assay Kit (ThermoFisher Scientific, Waltham, MA, USA), respectively. Library preparation was performed by using the Nextera XT DNA Library Prep Kit (Illumina, San Diego, CA, USA). The library generated was then diluted and sequenced with MiSeq Reagent Kit v2 (300-cycles) (Illumina, San Diego, CA, USA) on the MiSeq platform. The quality of raw sequences obtained from MiSeq run was first checked using FastQC (v 0.11.5) (Babraham Bioinformatics). The reads were aligned on reference sequence (GISAID accession ID: EPI_ISL_412973) using BWA-mem and rescued using Samtools alignment/Map (v 1.9) and bamtoFastq. Finally, the contigs were generated using SPAdes (v 3.12.0).

### HCQ Dose-Response Assessment

Vero E6 cells (4 × 10^5^ cells/mL) were seeded into 96 wells plates and treated with HCQ at different concentrations (1:3 serial dilutions, spanning from 10 to 0.1 μM). Cells were pre-treated with the drug for 1 h prior to virus infection at 37°C, followed by virus adsorption (50 TCID_50_/mL, 98 PFU/mL, SARS-CoV-2) for 1 h in the presence of the molecule. Then, the cells were washed with PBS and further cultured at 37°C with the molecule-containing medium for 72 h. Then, cytopathic effect (CPE) was assessed using a scoring system (0 = uninfected; 0.5 to 2.5 = increasing number/area of plaques; 3 = all cells infected) to evaluate treated and untreated cells. Infection control (score 3) was set as 0% infection inhibition, uninfected cells (score 0) as 100% infection inhibition. The whole surface of the wells was considered for the analysis (5x magnification). Cell supernatants were collected for real-time PCR (RT PCR) analysis. All conditions were tested in quadruplicate.

### HCQ Antiviral Effect Using Different Multiplicities of Infection (MOI)

Vero E6 cells (4 × 10^5^ cells/mL) were seeded into 96 wells plates and treated with 10 μM HCQ. In detail, cells were pre-treated with the drug for 1 h prior to virus infection at 37°C, followed by virus adsorption at different concentrations (0.01–1 MOI, SARS-CoV-2) for 1 h in the presence of the molecule. Then, the cells were washed with PBS and further cultured at 37°C with the molecule-containing medium for 72 h. Then, the cytopathic effect (CPE) was assessed using the scoring system above described, and results were normalized on virus infection control. Cell supernatants were collected for RT-PCR analysis. All conditions were tested in triplicate.

### XTT Assay for Determination of Cell Viability

Cell viability assay was performed using the Cell Proliferation kit II (XTT) (Roche Diagnostics, Merck). Briefly, the tetrazolium salt 2,3-bis-(2-methoxy-4-nitro-5-sulfophenyl)-2H-tetrazolium-5-carboxanilide (XTT) is cleaved by viable cells to form an orange formazan dye that can be quantified photometrically at 450 nm. Before the assay, Vero E6, Caco-2, and Calu-3 cells (4 × 10^5^ cells/mL) were cultured in 96-well plates for 24 h. The culture medium was replaced by medium containing 10 μM HCQ following *full-time* experimental setting, and cells were incubated for 72 h. XTT was added to each well and the plates were incubated for an additional 4 h. The optical density was measured at 450 nm (reference wavelength−650 nm) using a Multiskan GO plate reader (Thermo Scientific Instruments). For quantifications, the background levels of media without cultured cells were subtracted.

### Time-of-Addition Experiments of HCQ

Vero E6 cells (4 × 10^5^ cells/mL) were seeded into 96 wells plates and treated with HCQ (10 μM) at different stages of virus infection. For *full-time* treatment, cells were pre-treated with the drug for 1 h prior to virus infection at 37°C, followed by virus adsorption for 1 h in the presence of the molecule. Then, cells were washed with PBS, and further cultured at 37°C with the molecule-containing medium until the end of the experiment. For *pre-adsorption* treatment, the agent was added to the cells for 1 h at 37°C before virus infection and maintained during virus adsorption. Then, the mixture was replaced with fresh medium without molecule till the end of the experiment. For *post-adsorption* assays, the drug-containing medium was added to cells only after virus adsorption and maintained until the end of the experiment. BFLA1 (100 nM) was tested as control of inhibition of viral infectivity at a phagolysosome level only in a *pre-adsorption* treatment, alone or in combination with *pre-adsorption* HCQ treatment. Uninfected cells were included in all experimental settings to exclude possible drug-toxicity CPE. For all the experimental groups, cells were infected with 50 TCID_50_/mL SARS-CoV-2, and absorption was performed for 1 h at 37 or 4°C. The *full-time* experimental setting was performed also using Caco-2 and Calu-3 cells. Live images were acquired (Olympus CKX41 inverted phase-contrast microscopy), CPE was assessed as described above and cell supernatants were collected for RT-PCR analysis at 72 hpi. All conditions were tested in quadruplicate.

### Viral RNA Extraction and Real-Time RT-PCR

Viral RNA was purified from 140 μL of cell culture supernatant using the QIAamp Viral RNA Mini Kit (QIAGEN), following the manufacturer's instructions. Subsequently, the purified RNA was used to perform the synthesis of the first-strand cDNA, using the SuperScript™ First-Strand Synthesis System for RT-PCR (Thermo Fisher Scientific), following the manufacturer's instruction. Real-time PCR, using SYBR® Green dye-based PCR amplification and detection method, was performed in order to detect the cDNA. We used the SYBR™ Green PCR Master Mix (Thermo Fisher Scientific) the forward primer N2F: TTA CAA ACA TTG GCC GCA AA, the reverse primer N2R: GCG CGA CAT TCC GAA GAA, and the following PCR conditions: 95°C for 2 min, 45 cycles of 95°C for 20 s, annealing at 55°C for 20 s and elongation at 72°C for 30 s, followed by a final elongation at 72°C for 10 min (Hirotsu et al., 2020). RT-PCR was performed using the ABI-PRISM 7900HT Fast Real-Time instruments (Applied Biosystems) by using optical-grade 96-well plates. Samples were run in duplicate in a total volume of 20 μL.

### Statistical Analysis

CPE observed for different experimental settings using HCQ and BFLA1, alone or in combination, were normalized to corresponding virus infection control. RT-PCR results were analyzed calculating Delta (Δ) Ct as the difference between Ct values obtained for experimental settings and infection control. Then, two-way ANOVA and Tukey's multiple comparisons (for Caco-2 and Calu-2 experiments and virus curve for testing HCQ susceptibility) or Sidak's multiple comparisons (for HCQ dose-response curve, time of addiction experiments and XTT cell viability evaluation) test were performed for the evaluation of CPE scoring and Ct differences (GraphPad Prism 8). EC_50_ was calculated using non-linear regression with least squares regression as a fitting model (GraphPad Prism 8).

## Results

### Virus Isolation and Sequencing

Virus isolation was achieved after <72 h. At 48 hpi the cytopathic effect (CPE) was already evident on Vero E6 cells. NGS analysis was performed by Illumina MiSeq obtaining the whole genome sequence of the cultured isolate hCoV-19/Italy/UniSR1/2020 (GISAID accession ID: EPI_ISL_413489). Compared to the first Italian isolate sequenced (hCoV-19/Italy/CDG1/2020), that diverged from the hCoV-19/Wuhan/WIV04/2019 reference strain at position 241, 3,037, 14,408 and 23,403, two more nucleotide mismatches were identified, G187A and C6956A. G187A is located in the 5′UTR, while C6956A is a missense mutation causing the I2231L variation in the ORF1a polyprotein, specifically in the region that is part of the nsp3 membrane domain. No obvious consequence of the two polymorphisms can be drawn based solely on the sequence.

### HCQ Dose-Response Assessment

**Figure 1:**
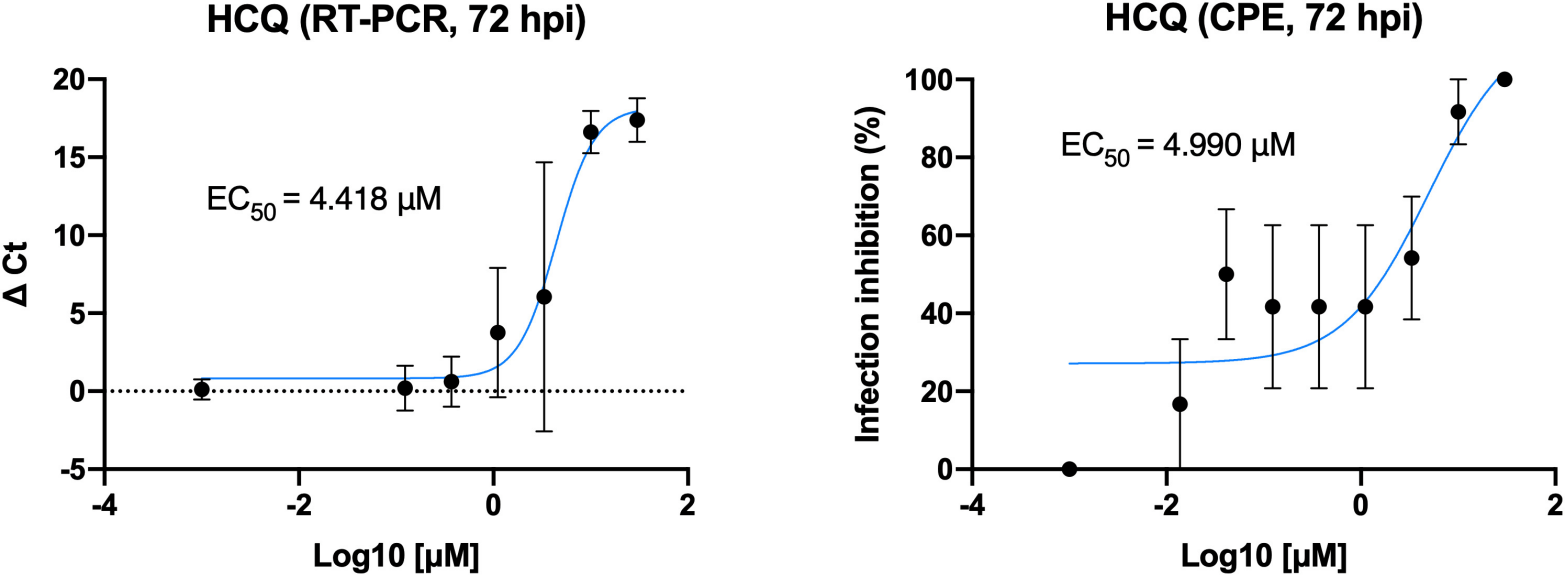
HCQ dose-response assessment. HCQ EC_50_ against SARS-CoV-2 was obtained by both CPE and RT-PCR analysis on results from *full-time* experimental setting on Vero E6 cells. Mean values and SD (for RT-PCR values) and SEM (for CPE values) are reported for all experimental replicates. All conditions were tested in quadruplicate and tested in duplicate in RT-PCR.

Different concentrations of HCQ were tested on Vero E6 to determine the effective concentration of the drug against SARS-CoV-2 *in vitro* infection (Figure 1). EC_50_ resulted from both CPE and RT-PCR analysis were 4.418 and 4.990 μM, respectively.

### HCQ Antiviral Effect Using Different Multiplicities of Infection (MOI)

**Figure 2:**
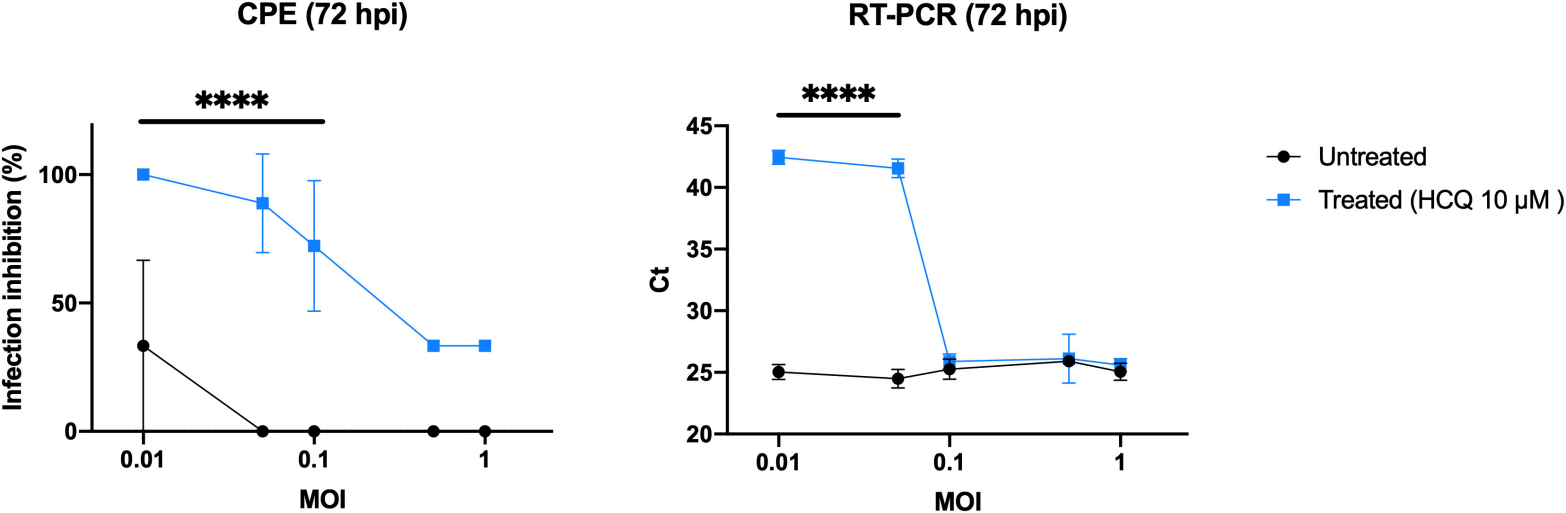
HCQ antiviral effect using different multiplicities of infection (MOI). CPE analysis resulted in a statistical difference between treated and untreated cells (****P < 0.0001) when using 0.1 MOI or less, RT-PCR only from 0.05 MOI to decrease (****P < 0.0001).

As EC_50_ analysis resulted in a low HCQ effective dose, it was tested using different viral loads to better define the optimal conditions to complete the preliminary setting for subsequent experiments (Figure 2). CPE analysis resulted in a statistical difference between treated and untreated cells (*P* < 0.0001) when using 0.1 MOI or less, RT-PCR only from 0.05 to decrease (*P* < 0.0001).

### Cell Viability Evaluation

**Figure 3:**
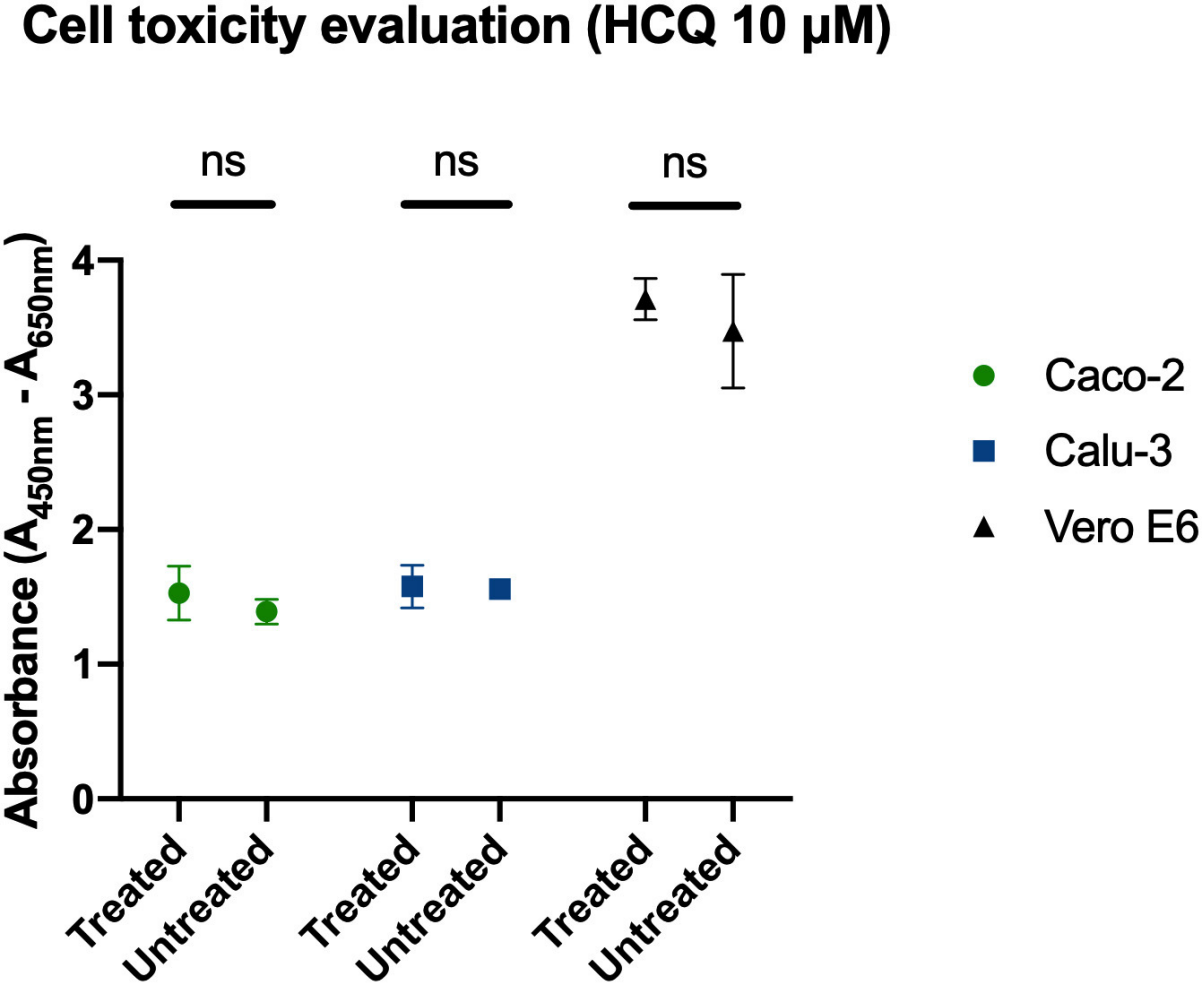
Determination of cell viability with HCQ treatment. *Full-time* treatment of Caco-2, Calu-3, Vero E6 cells with 10 μM HCQ does not affect cell viability. Mean and SD of optical density, ns = *P* > 0.05.

XTT assay was performed to determine cell tolerability to HCQ treatment, and 10 μM of the molecule tested in *full-time* treatment resulted in no drug-related toxicity on all three cell lines tested in the study (Figure 3).

### Time-of-Addition Experiments of HCQ and BFLA1

**Figure 4:**
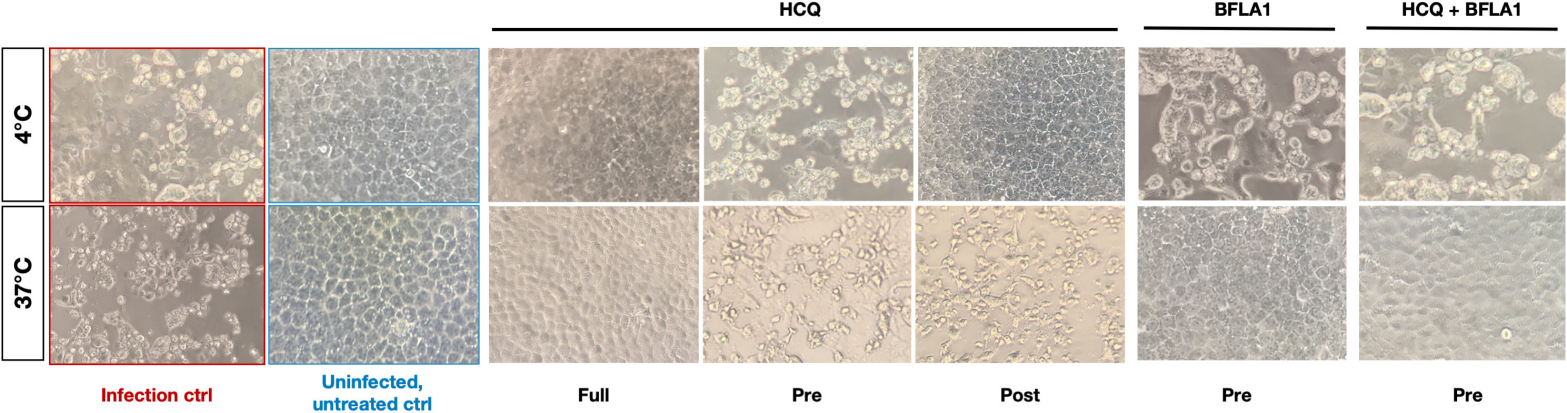
CPE on infected cells treated with HCQ in different experimental settings. Bright-field microscopy images (20x magnification, 72 hpi) of representative CPE of hCoV-19/Italy/UniSR1/2020 (GISAID accession ID: EPI_ISL_413489) isolate detected on both untreated cells (Infection control, red border) and treated cells with different experimental settings of HCQ treatment. Untreated, uninfected control show the proper cell morphology (blue border). Virus adsorption was performed at 4 and 37°C.

**Figure 5:**
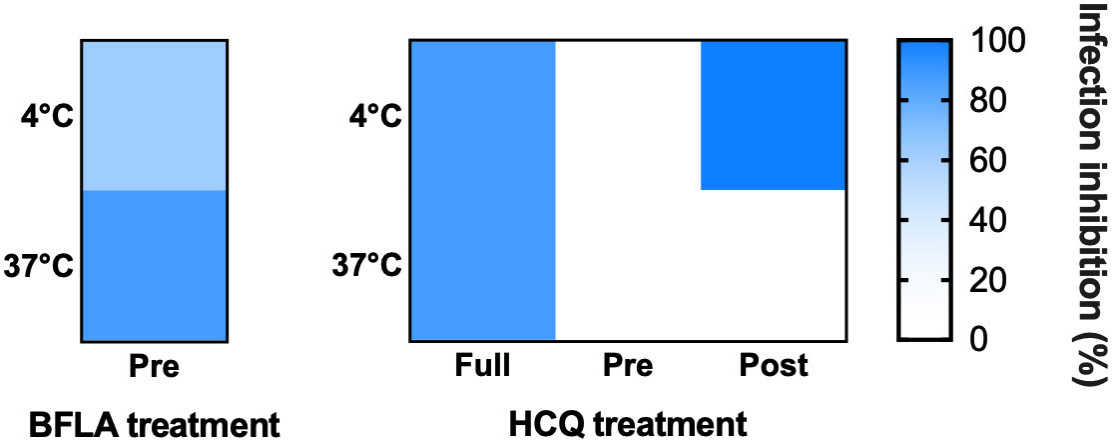
HCQ CPE reduction on VeroE6 cells infected with SARS-CoV-2. CPE was assessed using a scoring system (0 = uninfected; 0.5 to 2.5 = increasing number/area of plaques; 3 = all cells infected) to evaluate treated and untreated cells. Infection control (score 3) was set as 0% infection inhibition, uninfected cells (score 0) as 100% infection inhibition. The whole surface of the wells was considered for the analysis (5x magnification). Blue gradient indicates the reciprocal of CPE detected in treated cells compared to virus infection control (white color corresponds to 100% CPE). Protection levels are indicated by color gradient: white corresponds to no protection, dark blue shows full protection. BFLA1 treatments are reported as experimental control of virus fusion process inhibition. Virus adsorption was performed at 4 and 37°C.

The two molecules were tested using different experimental protocols, and virus adsorption was performed at both 37 and 4°C. CPE was assessed at 72 hpi (Figures 4, 5). Virus infection positive control showed marked effects on cell morphology at 37°C as well as 4°C adsorption conditions. HCQ was effective in *full-time* treatment at both adsorption temperatures, and in *post-adsorption* treatment only when the virus was added to cells for 1 h at 4°C. The molecules did not show the same degree of protection from CPE in *pre-adsorption* treatment at both adsorption temperatures, as well as in *post-adsorption* treatment at 37°C adsorption. BFLA1 was tested as control of inhibition of viral infectivity at a phagolysosome level in a *pre-adsorption* treatment, showing full CPE protection at 37°C virus adsorption, while only partial protection was observed when the virus was added to cells at 4°C (Figures 4, 5). No drug-related cytotoxic effect was observed on uninfected cells, in all experimental settings.

### RT-PCR Analysis of HCQ Experimental Settings

**Figure 6:**
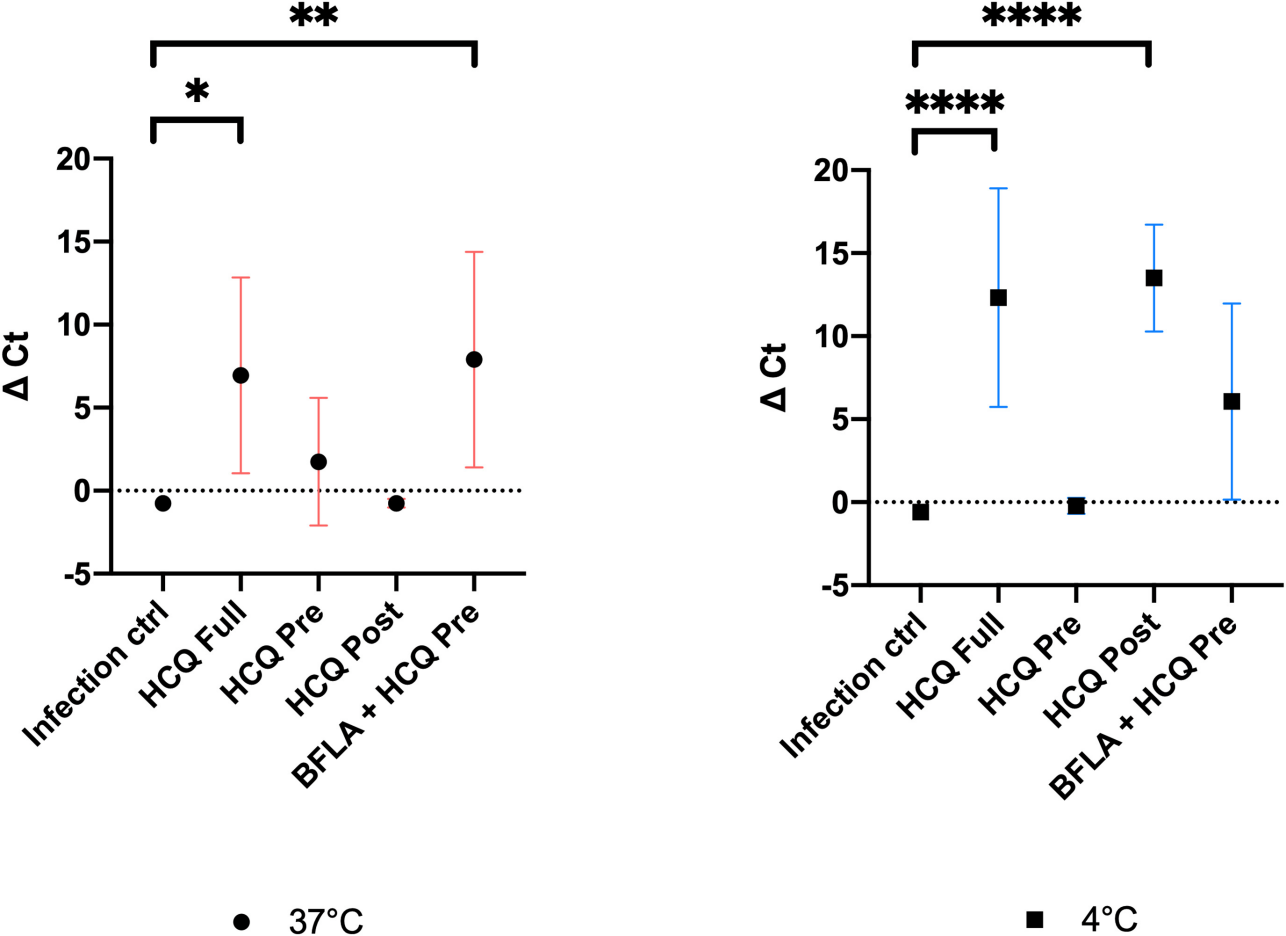
RT-PCR analysis of cell supernatants of different experimental settings. Ct levels are inversely proportional to the amount of target nucleic acid in the sample (the lower the Ct level the greater the amount of virus within the tested supernatant). Graphs show virus adsorption at 37 and at 4°C. Delta (Δ) Ct are represented in y axis. Median values for all experimental replicates, tested each one in duplicate in RT-PCR, and 95% IC range reported with error bars (*P < 0.05; **P < 0.01; ****P < 0.0001). All conditions were tested in quadruplicate.

**Figure 7:**
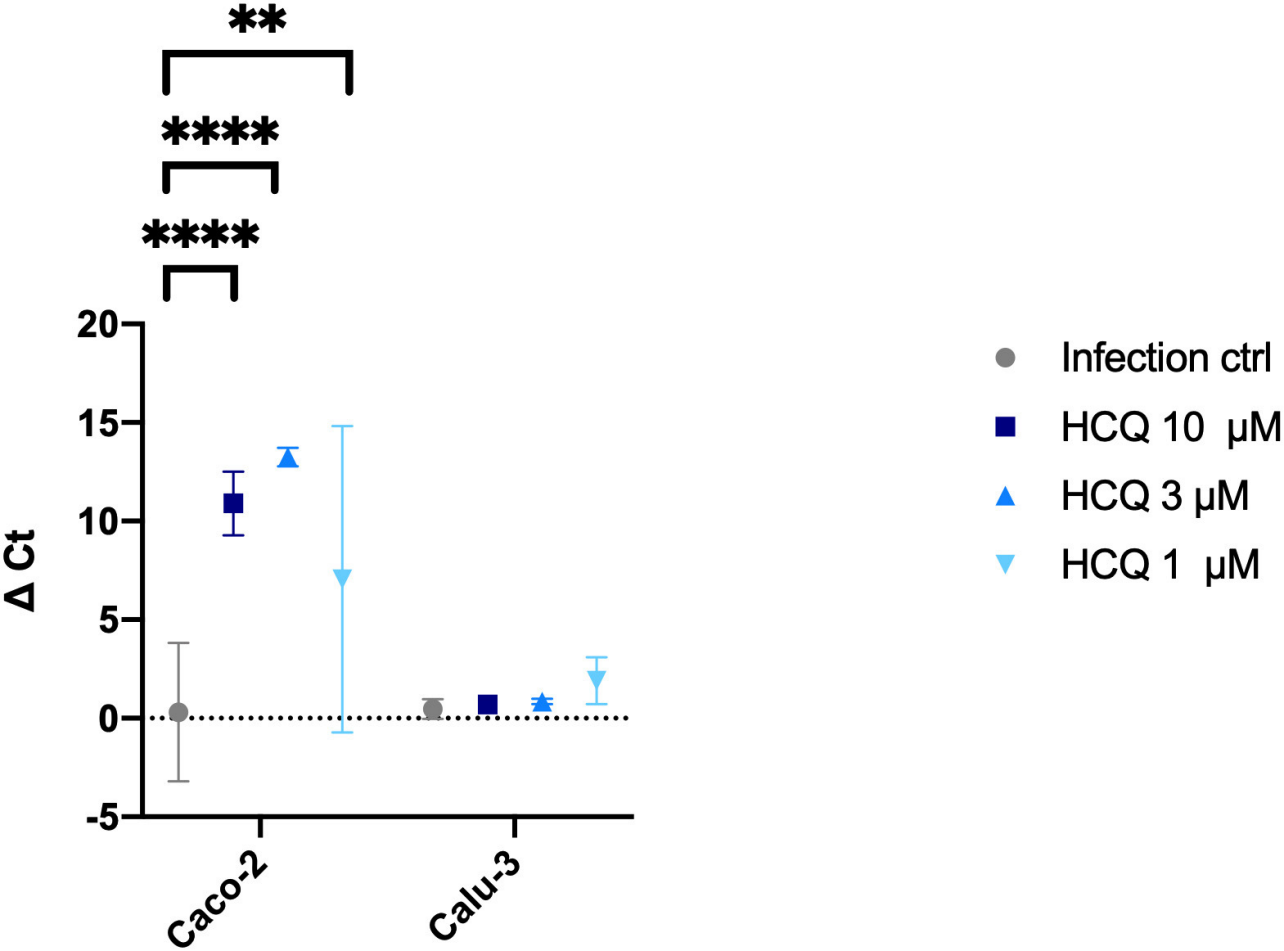
RT-PCR analysis of HCQ treatment of different cell lines. Three different concentrations of HCQ were tested on Caco-2 and Calu-3 cells using *full-time* experimental setting. Graph show virus adsorption at 37°C. Delta (Δ) Ct are represented in y axis. Median values for all experimental replicates, tested each one in duplicate in RT-PCR, and 95% IC range reported with error bars (**P < 0.01; ****P < 0.0001). All conditions were tested in quadruplicate.

Cell supernatants of different experimental settings were collected and analyzed by RT-PCR, and results confirmed CPE data analysis (Figure 6). In detail, a significant statistical difference of ΔCt was observed with HCQ *full-time* treatment compared to infection control, both at 37°C (*P* < 0.05) and 4°C (*P* < 0.0001) virus adsorption, while HCQ *post-adsorption* treatment was effective (*P* < 0.0001) only when the virus was added to cells at 4°C. Interestingly, BFLA1 addition to HCQ *pre-adsorption* treatment resulted in a significant statistical difference of ΔCt when compared to infection control at 37°C virus adsorption. When tested on different cell lines, HCQ resulted extremely effective on Caco-2 cells (*P* < 0.0001 for the higher tested concentrations, *P* < 0.01 for the minor one), while a non-statistical difference was observed when tested on Calu-3 cells (Figure 7).

## Discussion

In the lack of SARS-CoV-2-specific drugs, it is extremely important to evaluate the clinical potential of drug-repurposing to face the current pandemic (Yao et al., 2020). HCQ has gained the attention of the scientific and medical community based on previous *in vitro* data on similar viruses (SARS and MERS) and on preliminary reports discussing its possible clinical effectiveness in therapy or prophylaxis based on observations performed on Vero E6-based infection models (Colson et al., 2020; Gao et al., 2020; Gautret et al., 2020). In this atypical context, in which on-field medicine often anticipates experimental laboratory pre-clinic, there is an urgent need for prompt experiments addressing specific clinical questions. For example, it is not clear what is the best administration regimen to maximize possible HCQ anti-viral effectiveness in COVID-19 patients. In this regard, a recent study evaluated the direct anti-SARS-CoV-2 effect *in vitro* and, importantly, modeled its bioavailability at the lung level where it could maximally exert its antiviral activity. Based on a dosing regimen of 400 mg given twice daily for 1 day, followed by 200 mg twice daily for 4 more days, it also suggested the more useful drug concentrations to be used in clinically-oriented phenotypic laboratory assays (Liu et al., 2020; Wang et al., 2020; Yao et al., 2020).

On that basis, we evaluated the HCQ antiviral activity when administered before (*pre-adsorption*), after (*post-adsorption*), or before and after (*full-time*) virus adsorption to simulate, on Vero E6 cells, its possible prophylactic, therapeutic and prophylactic/therapeutic clinical use. Moreover, we first focused our attention on a single concentration (10 μM) easily achieved, well-tolerated and endowed with a strong antiviral activity (Yao et al., 2020). Moreover, to speculate on its possible mechanism of action, we also evaluated HCQ activity performing virus adsorption at 37 and 4°C. In fact, at 37°C the virus enters the cell in a more physiological context while, conversely, at 4°C virus it can dock to the cell receptor, but its internalization is much more limited.

In the prophylactic setting (*pre-adsorption*), 10 μM HCQ did not interfere effectively with the viral replicative cycle neither at 37°C nor at 4°C, as evidenced in the CPE and the RT-PCR analysis. Limited antiviral activity was also observed in the therapeutic setting (*post-adsorption*) but, interestingly, a higher HCQ antiviral activity was observed at 4°C, suggesting its predominant SARS-CoV-2 interference at the endosomal level (Vincent et al., 2005; Hu et al., 2020). Overall, and most importantly, these results suggest a limited activity of HCQ when administered only prophylactically or therapeutically.

On the contrary, significant antiviral activity was observed in the prophylactic/therapeutic (*full-time*) experimental setting both at 37 and 4°C, as evidenced both by CPE and RT-PCR analyses. This observation allows us to speculate on the need for a combined prophylactic and therapeutic clinical use of HCQ to maximize its antiviral effects. However, a possible bias of *in vitro* studies aimed at evaluating the antiviral activity of putative drugs can be represented by the MOI used for testing the inhibitory activity of drugs. That is why we used different MOI for testing the inhibitory activity of HCQ. Our data suggest how using MOI higher than 0.1 to perform *in vitro* testing can impact on the HCQ inhibitory capability.

We also determined, by CPE evaluation and RT-PCR analysis, the HCQ EC_50_ (4.418 and 4.990 μM, respectively) at 72 h post-infection. Moreover, to better translate to the clinic what observed on Vero E6, HCQ activity was assessed also on cells of human origin: the Caco-2 (colorectal adenocarcinoma epithelial-like cells) and the Calu-3 (lung adenocarcinoma epithelial cells). As observed for Vero E6 cells, no HCQ direct toxicity was observed for both Caco-2 and Calu-3. Interestingly, a dose-response effect of HCQ was appreciated only on Caco-2 cells. On the contrary, no significant reduction of viral RNA amount was appreciated on Calu-3 cells. This observation will deserve certainly further investigations focused on both mechanistic aspects of HCQ-mediated inhibition of SARS-CoV-2 and on the possible interpretation of clinical trial outcomes.

In the atypical scenario of an ongoing pandemic, pre-clinical medical research should be focused on simple and fast observations potentially useful for the prompt set up of clinical trials. However, all possible HCQ side effects already known (Chatre et al., 2018; Zhou et al., 2020) should be considered and evaluated also under the light of concerns regarding two clinical trials recently described and retracted (Mehra et al., 2020a,b; Science AAFTAO, 2020). As an example, our observation could be translated in a clinical study on extremely high-risk categories, such as health care workers, based on the prophylactic administration of HCQ followed by its therapeutic use in case of positivity to SARS-CoV-2.

## Data Availability Statement

Whole genome sequence data of hCoV-19/Italy/UniSR1/2020 have been uploaded on GISAID database (https://www.gisaid.org/) with the following accession ID: EPI_ISL_413489.

## Ethics Statement

The study involving human participants was reviewed and approved by Ospedale San Raffaele IRB in the COVID-19 Biobanking project. The patient from whose samples the virus was isolated provided written informed consent.

## Author's Note

This manuscript has been released as a pre-print at bioRxiv preprint. doi: 10.1101/2020.03.29.014407, Hu et al. (2020).

## Author Contributions

NC and NM conceived the study. EC, RD, and RF performed the experiments. NC, NM, EC, MCa, and MCl analyzed the data. NC, NM, EC, and RD wrote the manuscript. NC, NM, LD, RB, and MCl revised the manuscript. All authors contributed to the article and approved the submitted version.

## Conflict of Interest

The authors declare that the research was conducted in the absence of any commercial or financial relationships that could be construed as a potential conflict of interest.
